# Microbial Metabolic Pathways and the “Fermented Plant Foods—Human Health” Axis

**DOI:** 10.3390/foods10051105

**Published:** 2021-05-17

**Authors:** Raffaella Di Cagno, Pasquale Filannino, Marco Gobbetti

**Affiliations:** 1 Faculty of Sciences and Technology, Libera Università di Bolzano, 39100 Bolzano, Italy; marco.gobbetti@unibz.it; 2 Department of Soil, Plant and Food Science, University of Bari Aldo Moro, 70126 Bari, Italy

Plant matrices are widely recognized as valuable sources of several health promoting compounds. However, most of such plant beneficial compounds are encrypted within macromolecules and present low bioaccessibility and bioavailability.

In order to exert a health benefit, phytochemicals might need to be released from the plant matrix components and converted into accessible bioactive compounds. Their transition may be driven by several factors, including the microbial processing. Microbial metabolisms may also enrich plant substrates with functional microbial metabolites, and/or decrease the inherent content of anti-nutritional factors, with beneficial repercussions for human health [1].

To pursue the “fermented plant foods‒human health” axis, the fruitful utilization of microbial metabolisms has to go hand in hand with the product innovation, through the design of novel food formulations or enhancing the use of non-conventional plant matrices and food industry by-products. This Special Issue emphasizes the implementation of microbial metabolisms to boost the health-promoting properties of plant-based ingredients or foods through the fermentative processes. Lactic acid fermentation is the most implemented [1]. Nevertheless, bioprocessing through other microbial groups (e.g., yeasts) holds beneficial potentials deserving to be further investigated and exploited. Within the frame of unconventional matrices, bee-collected pollen (BCP) is attractive as a dietary supplement for humans. BCP may potentially supply a significant part of the required daily intake for sugars, proteins, and some vitamins and minerals, but it is not easily digestible by humans. More than 50% of BCP nutrients are not bioaccessible, due to the intine–exine complex of pollen grain wall. Therefore, BCP needs to be pre-treated before intake by humans. Among the proposed biotechnological options, fermentation is one of the most appealing opportunities [2]. Focusing on the fast-growing segments of functional beverages, M’hir et al. [3] designed a novel carob-based kefir-like beverage, fortified with whey permeate and oat flour. Their study addressed several hot topics, including the food by-products exploitation, and the interactions between different food matrixes. In particular, the response surface method was implemented to investigate the effect of ingredients on fermentation parameters, total phenolics content, radical scavenging activity, and overall acceptability. Emerging studies indicate a great interest in non-dairy fermented juices for probiotics delivery. Xu et al. [4] evaluated the feasibility of producing a symbiotic beverage based on carrot juice fermented with probiotic *Lactobacillus gasseri* strains. Carrot was chosen as a substrate due to the low acidity and the richness in sugars, vitamins, minerals, and phytochemicals, such as carotenoids and polyphenols. The assumption was that the fermentation of a sugar rich substrate with *L. gasseri* strains may enable the synthesis of prebiotic fructans.

Bontsidis et al. [5] focused their study on the probiotic viability and chemical changes occurring during the cold storage of chokeberry juice fermented by *Lactobacillus paracasei* SP5. This latter is a potential probiotic strain recently isolated from kefir grains.

Among plant matrices, legumes were proposed as irreplaceable ingredients for making various baked goods and pasta, due to their potential to improve protein quality in cereal products. In parallel, fermentation was suggested as a valuable biotechnology to overcome the poor rheological properties and the content of antinutritional compounds of legume flours. Hoehnel et al. [6] fermented two faba bean flours with different protein contents with the multifunctional strain *Leuconostoc citreum* TR116. Their study combined the fundamental characterization of faba bean fermentation with an in-depth evaluation of the fermented ingredient application in bread, obtaining results highly relevant for advancing the protein transition. De Pasquale et al. [7] applied a black chickpea flour pre-fermented with *Lactiplantibacillus plantarum* T0A10 for the fortification of pasta. After fermentation, the authors evaluated the changes of nutritional features, antioxidant activity, and phenolic profile in black chickpea flour. Moreover, they explored the impact of fermented black chickpea on the technological, nutritional, functional, and sensory properties of fortified pasta.

Fermentation processes may detoxify or degrade antinutritional compounds that are inherently present in cereal grains, other than in legumes. Wheat germ agglutinin (WGA) and α-amylase/trypsin inhibitors (ATI) are wheat components related to health disorders. Tovar et al. [8] investigated the changes of WGA level attributable to the dough fermentation with *Fructilactobacillus sanfranciscensis* DSM20451, or its isogenic derivative lacking glutathione reductase activity, or *Latilactobacillus sakei* TMW1.22, even in the presence of fungal protease. Their experimental approach was useful to evaluate whether acidification and/or the reduction of disulfide bonds contribute to the degradation of WGA. The fate of ATI during the sourdough or bakery-yeast bread-making was investigated by Huang et al. [9], who described the molecular changes of ATI as consequence of fermentation, proteolytic, and thermal processes.

Whether lactic acid bacteria and yeasts are useful drivers for the bioconversion of plant compounds, not all species and strains are equipped with the required portfolio of enzymes. Different plant niches harbor a huge genetic potential constituted by specialized and high performing microbial strains, but hitherto unexploited. For instance, *Liquorilactobacillus hordei* is a lactic acid bacterium strongly adapted to sucrose-rich habitats and dominating the water kefir consortium. Recently, this species gained interest due to its dextran-producing abilities. Bechtner et al. [10] deeply investigated the physiological response of *L. hordei* TMW 1.1822 to sucrose compared to glucose. Label-free quantitative proteomics allowed to detect the differential expression of 53 proteins within *L. hordei* proteomes, mostly associated with carbohydrates metabolism. Their study highlighted metabolic pathways that may occur in addition to or in competition with dextran formation, and extended the knowledge on *L. hordei* exploitability and its ecological role during water kefir fermentation. Acin-Albiac et al. [11] proposed an innovative phenomic approach to describe the metabolism drift of *Lactiplantibacillus plantarum* and *Leuconostoc pseudomesenteroides* induced by lignocellulosic-derived substrates originating from brewers’ spent grain (BSG), compared to a conventional growth medium. BSG constitute the most abundant by-product generated in the beer-brewing process and represent a current challenge within cereal by-products valorization due to the complexity of its indigestible polymers and anti-nutritional factors. Author focused their study on the phospho-β-glucosidase activities, which are involved in the metabolism of carbohydrates and the release of phenolic compounds from their β-D-glycosylated precursors.

## Conclusions and Future Outlook

This collection of works aimed to offer an interesting overview, albeit incomplete, of the huge and not properly exploited microbial potential, implementable to boost and/or exploit the health-promoting properties of plant-based matrices. Much of the future efforts should focus on the reconstruction of the regulatory network underlying the microbial in situ behavior, as well as on the search of tailored strains for the diverse intrinsic and extrinsic conditions distinguishing each plant matrices processing.

## Data Availability

Data is contained within the article or supplementary material published in the Special Issue.
